# Prophage Spontaneous Activation Promotes DNA Release Enhancing Biofilm Formation in *Streptococcus pneumoniae*

**DOI:** 10.1371/journal.pone.0015678

**Published:** 2010-12-20

**Authors:** Margarida Carrolo, Maria João Frias, Francisco Rodrigues Pinto, José Melo-Cristino, Mário Ramirez

**Affiliations:** 1 Instituto de Microbiologia, Instituto de Medicina Molecular, Faculdade de Medicina, Universidade de Lisboa, Lisboa, Portugal; 2 Centro de Química e Bioquímica, Faculdade de Ciências, Universidade de Lisboa, Campo Grande, Lisboa, Portugal; Institut Pasteur, France

## Abstract

*Streptococcus pneumoniae* (pneumococcus) is able to form biofilms *in vivo* and previous studies propose that pneumococcal biofilms play a relevant role both in colonization and infection. Additionally, pneumococci recovered from human infections are characterized by a high prevalence of lysogenic bacteriophages (phages) residing quiescently in their host chromosome. We investigated a possible link between lysogeny and biofilm formation. Considering that extracellular DNA (eDNA) is a key factor in the biofilm matrix, we reasoned that prophage spontaneous activation with the consequent bacterial host lysis could provide a source of eDNA, enhancing pneumococcal biofilm development. Monitoring biofilm growth of lysogenic and non-lysogenic pneumococcal strains indicated that phage-infected bacteria are more proficient at forming biofilms, that is their biofilms are characterized by a higher biomass and cell viability. The presence of phage particles throughout the lysogenic strains biofilm development implicated prophage spontaneous induction in this effect. Analysis of lysogens deficient for phage lysin and the bacterial major autolysin revealed that the absence of either lytic activity impaired biofilm development and the addition of DNA restored the ability of mutant strains to form robust biofilms. These findings establish that limited phage-mediated host lysis of a fraction of the bacterial population, due to spontaneous phage induction, constitutes an important source of eDNA for the *S. pneumoniae* biofilm matrix and that this localized release of eDNA favors biofilm formation by the remaining bacterial population.

## Introduction

Biofilms, the most frequently encountered physiological form adopted by microorganisms, are surface-adapted communities that constitute a protected mode of bacterial growth allowing survival in hostile environments [1]–[4]. Recent studies demonstrated the potential of *Streptococcus pneumoniae* to produce biofilms *in vivo* [5], [6]. Pneumococcal biofilms were indeed detected on affected tissues in patients with chronic rhinosinusitis [5], children with otitis media [6], as well as a chinchilla model of otitis [7]. The presence of these communities at the site of infection implicates them in these disorders, although their significance in the infection process is a matter of debate. Two recent studies have failed to find an association between the ability to form biofilms and whether the isolates had been recovered from asymptomatic carriers or caused invasive infections [8], [9]. Moreover, a mouse model of invasive infection failed to show any association between the capacity to cause bacteremia and the ability of the strains to form robust biofilms [8]. Although these two studies question the role of biofilms in determining the invasive potential of pneumococci, the transcriptional profile of several known virulence-related genes in *S. pneumoniae* isolated from lungs and brains of infected mice is similar to that in biofilms formed *in vitro*, suggesting a possible biofilm-like state of *S. pneumoniae* associated with tissues [10]. In addition, a link was established between pneumococcal biofilm formation and the asymptomatic colonization of the nasopharynx [11], the most frequent state of pneumococci. Overall, these studies highlight the importance of studying *S. pneumoniae* biofilms, particularly of identifying the factors that influence the formation of these structures.

Bacterial biofilms are encased within an extracellular matrix consisting of polysaccharides, proteins and nucleic acids [2]. Although polysaccharides and proteins are important components, the role of extracellular DNA (eDNA) as a critical element of the matrix is increasingly recognized, both in providing structural stability as well as protection against antimicrobial agents [12]–[15]. In Gram-positive bacteria, such as *Enterococcus faecalis* and *Staphylococcus epidermidis*, autolysins (bacterial murein hydrolases) were recently implicated in biofilm formation, apparently by mediating bacterial lysis with the consequent release of eDNA [15]–[18]. Pneumococcal cells are characterized by the presence of a major autolysin LytA, an N-acetyl-muramyl-L-alanine amidase, which is responsible for the unusual property of massive cellular lysis displayed in the stationary phase of liquid cultures [19]. Furthermore, cell lysis dependent on LytA was also detected upon competence development, which results in DNA release into the medium [20], [21]. The observation that *S. pneumoniae* biofilm formation is influenced by the presence of eDNA [22], [23] and that LytA mutants have a decreased capacity to form biofilms [23], hints that LytA-induced pneumococcal lysis could be related to biofilm formation through the release of eDNA.

In addition to autolytic events, cell lysis in *S. pneumoniae* can also be mediated by lysogenic phages, which have a high prevalence (76%) in isolates associated with infection [24], [25]. During lysogeny, the prophage is integrated in the bacterial chromosome being replicated as part of the hosts genome. Upon induction, the repressed lysogenic state shifts to lytic growth with the production of viral particles and subsequent phage mediated host lysis to release the phage progeny [24], [26]. It was recognized early that free phages can be found in cultures of lysogenic bacteria in the absence of a known inducing agent, indicating that some prophages spontaneously enter the lytic cycle [27]. Spontaneous phage induction seems to be a common feature of lysogeny, being non-specific of the phage or the bacterial host, although the factors that promote spontaneous induction, either *in vitro* or *in vivo*, are poorly understood. Recent studies showed that this natural phenomenon may contribute to pathogenicity in *Salmonella* [28], increasing the awareness of the potential importance of lysogeny in the context of infection. This spontaneous phage release occurs obviously at low levels, and the phage titer observed is orders of magnitude less than the one produced when the same bacteria are treated with an inducing agent [26], [29], [30].

Whether agent-induced or spontaneous, it was believed that phages of *S. pneumoniae* relied exclusively on their own lysins to hydrolyze host cell wall peptidoglycan and release the phage progeny [31]. Recently, it was shown that pneumococcal lysogenic phages achieve an optimal exit strategy by orchestrating the coordinated action of the phage-encoded lysin and the bacterial major autolysin LytA [32].

Inevitably, prophage activation results, through bacterial lysis, in the release of the cellular components to the extracellular medium. Since eDNA is increasingly recognized as a critical element for biofilm formation, we hypothesized that spontaneous induction of lysogenic phages could have a positive effect on pneumococcal biofilms. To test this, we have evaluated biofilm formation and eDNA release of isogenic strains differing in carriage of a prophage and having functional or being deleted in the major phage and bacterial lysins.

## Results

### Lysogenic phages enhance biofilm development

**Figure 1:**
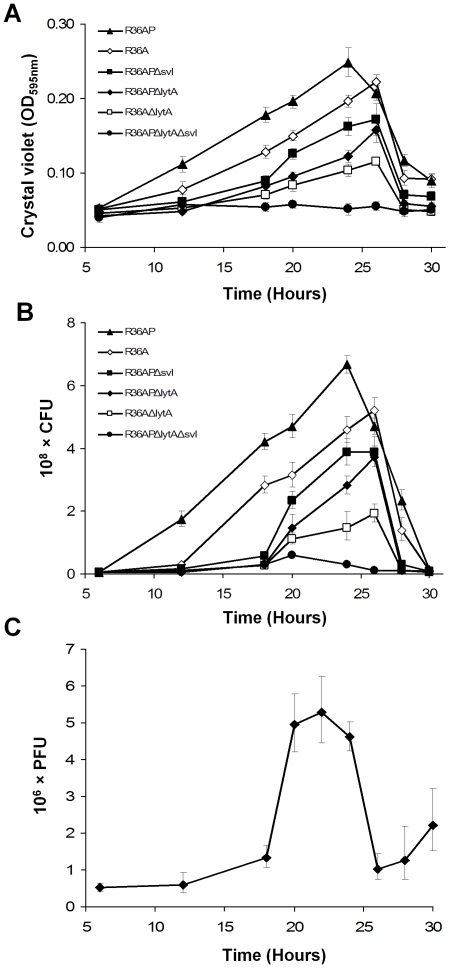
Effect of lysogeny and phage induction in *Streptococcus pneumoniae* biofilm development. **A**) Biofilm development monitored as biomass from 6 h to 30 h. R36A non-lysogenic strain; R36AP lysogenic derivative of R36A; R36APΔ*svl*, R36APΔ*lytA*, R36AΔ*lytA* and R36APΔ*lytA*Δ*svl* are mutants in which the phage lysin (Svl), the bacterial autolysin (LytA) or both were deleted. Results are an average of 9 independent replicates. **B**) Biofilm development monitored as CFUs from 6 h to 30 h. The strains are the same indicated in panel A. Results are an average of 6 independent replicates. **C**) The presence of phage in the R36AP biofilm was determined by the production of plaques on R36A. PFUs were determined throughout biofilm development from 6 h to 30 h. Results are an average of 2 to 7 independent replicates for each time point. In all panels error bars represent 95% confidence intervals for the sample mean.

**Figure 2:**
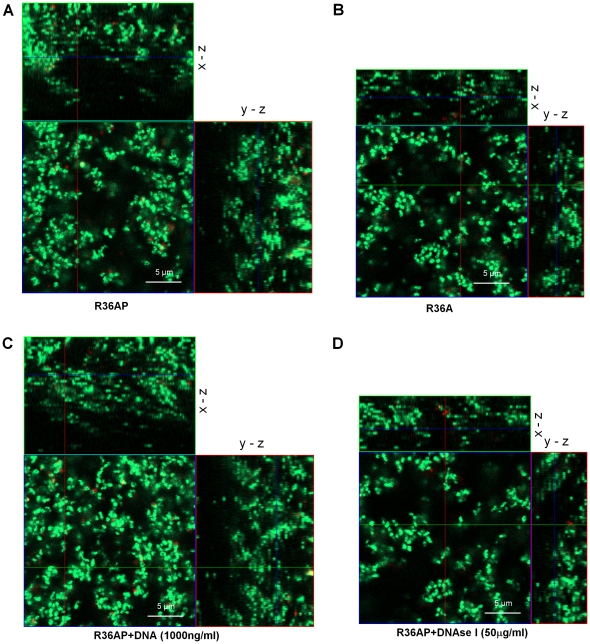
Confocal laser scanning microscopy images of R3A6P and R36A biofilms. Staining was done with Syto 9/PI (Live/Dead BacLight Bacterial Viability kit) and images were acquired at 630×amplification. Only live cells internalize Syto 9 (fluorescing green) whereas dead cells allow the uptake of PI (fluorescing red). The large images are optical sections of top views and the small images to the right and above are optical sections of side views. The depth of the biofilm is indicated by the height of the *z*-stack. The inset scale bar represents 5 µm. **A**) Biofilm formed by the lysogenic strain R36AP. **B**) Biofilm of the non-lysogenic strain R36A. **C**) The biofilm was grown in the presence of salmon sperm DNA at 1000 ng/ml. **D**) The biofilm was grown in medium supplemented with DNase I at 50 µg/ml. In all panels the results are representative images of 3 independent experiments and biofilm growth was evaluated at 24 h.

In order to evaluate the impact of lysogeny in biofilm formation a well established *in vitro* system, based on an abiotic surface as the growth substrate, was used allowing proper investigation of the initial stages of biofilm formation [8]–[10], [22], [23]. We started by monitoring biofilm development of the isogenic pair of *S. pneumoniae* strains R36A and R36AP, which differ only in the presence of a prophage (R36AP is a lysogen of phage SV1). Biofilm growth was followed at specific time points between 6 h and 30 h of incubation by biomass quantification and viable cell counts (Fig. 1A and 1B). The evaluation of cell viability by CFUs was consistent with biomass quantification obtained by crystal-violet staining. The biofilm of the lysogenic strain R36AP reaches its maximal development at 24 h and from that time onwards a decrease in biomass occurs. We reasoned that this decrease is inherent to the experimental conditions used, probably due to nutrient depletion, accumulation of toxic substances or intrinsic properties of the biofilm. In contrast, for the wild type non-lysogenic strain R36A the highest biofilm mass values are registered at 26 h, decreasing afterwards in a behavior similar to that of strain R36AP. This observation is consistent with a slower biofilm growth of strain R36A, resulting in delayed development. The lysogenic strain showed improved biofilm growth at all time points and also a higher maximal biofilm mass than its non-lysogenic parent. In agreement with these findings, images of CLSM show denser and thicker biofilms for R36AP (Fig. 2A and 2B). Since the lysogen R36AP is indistinguishable from its parental strain R36A in planktonic growth [32], the differences observed must be attributed to the influence of the lysogenic phage on biofilm formation.

### Spontaneous prophage induction enhances biofilm development due to host lysis

It was previously shown that the main pneumococcal autolysin LytA is important in normal biofilm development since its inactivation resulted in diminished biofilm formation, possibly by a mechanism dependent on its regulated lytic activity [23]. Thus, autolytic events may be helpful in the establishment of robust *S. pneumoniae* biofilms. It is well known that spontaneous phage induction results in the lysis of a fraction of the bacterial population [29] and we speculated that such induction could also occur within pneumococcal biofilms. Accordingly, the enhanced biofilm formation of the lysogenic strain R36AP could be explained by limited phage triggered lysis. To test this hypothesis, we compared biofilm development of the lysogenic strain R36AP to that of the derived mutants for phage lysin Svl (strain R36APΔ*svl*), bacterial autolysin LytA (R36APΔ*lytA*) or both lysins (R36APΔ*lytA*Δ*svl*). As shown in Fig. 1A and 1B biofilm growth is significantly impaired in the absence of the phage lysin with a shift in the biofilm biomass peak from 24 h to 26 h, analogous to the growth pattern observed for the non-lysogenic strain (R36A). A similar behavior was observed for the lysogen in the absence of the bacterial autolysin. In fact, the presence of at least one lysin is essential, as the double mutant was largely deficient in biofilm formation. Accordingly, the non-lysonenic R36AΔ*lytA* strain is also severely impaired in biofilm formation, supporting an important role of bacterial lysis in biofilm formation. This inability to form biofilms is not due to a growth defect since all mutants presented identical planktonic growth to the parent lysogen [32]. These results are consistent with the hypothesis that the positive impact of prophages in pneumococcal biofilm development is due to spontaneous induction of the lytic cycle resulting in cell lysis.

In order to confirm if phage induction was indeed occurring in the biofilm, we measured the phage particles released during biofilm development of strain R36AP by determining the number of PFUs throughout biofilm growth (Fig. 1C). We observed the presence of phages in the biofilm at all time points, indicating that spontaneous phage induction is occurring continuously and paralleling the increase in viable cells. A substantial increase in the number of PFUs coincides with the peak of biofilm development (Fig. 1A and 1B), indicating increased phage induction at the later stages of biofilm formation. This higher phage induction is not due to a massive triggering of the phage lytic cycle related to this stage of biofilm growth since no increase of PFUs concomitant with biofilm dispersal was observed. Altogether, these results strongly support a role of spontaneous phage induction during biofilm development in the different phenotype shown by lysogenic strains.

### Released eDNA through phage-mediated lysis is a key factor for biofilm enhancement

Extracellular DNA is an essential matrix component produced by many bacterial species during biofilm development [13], [15], [16], [33], [34]. Therefore, we hypothesized that in *S. pneumoniae* phage-mediated lysis of a fraction of the bacterial population within the biofilm could provide an extra source of eDNA for incorporation in the biofilm matrix.

**Figure 3:**
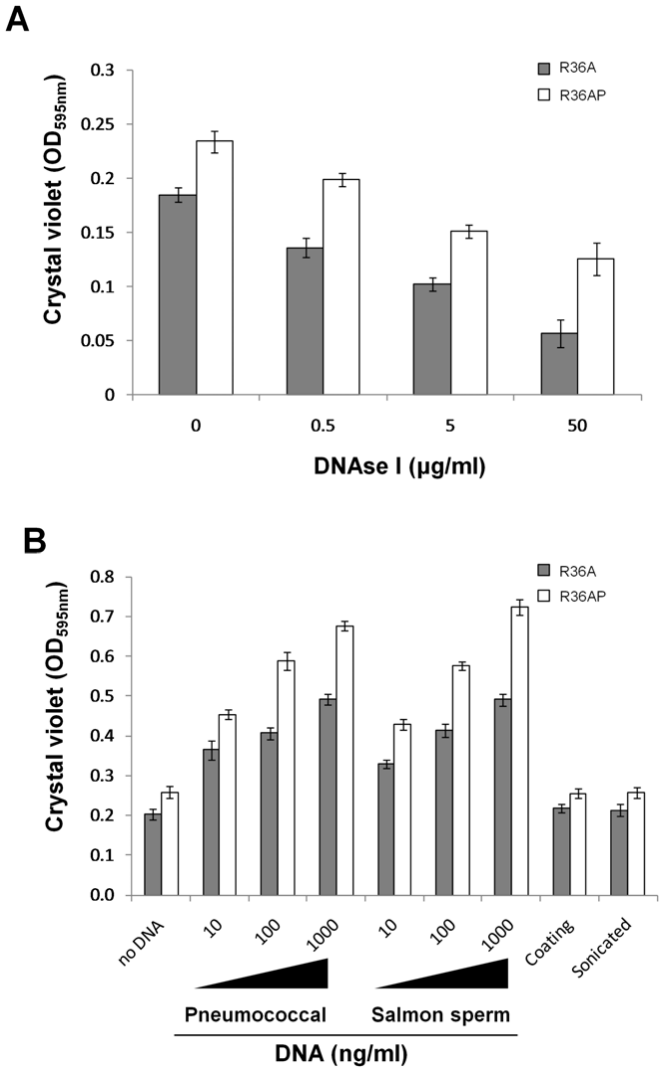
Effect of DNAse I and DNA on biofilm mass. **A**) The lysogenic strain R36AP and its non-lysogenic parent R36A were exposed from seeding to DNAse I at final concentrations of 0.5, 5 and 50 µg/ml. Biofilm mass was quantified after 24 h of incubation. **B**) R36AP and R36A were exposed from seeding to DNA from R36A or salmon sperm at final concentrations of 10, 100 and 1000 ng/ml. In separate experiments, the effect on biofilm development of coating the wells with 1000 ng/ml of R36A DNA prior to seeding and the addition of sonicated R36A DNA at 1000 ng/ml since the time of seeding was also determined. Biofilm mass was assessed at 24 h of incubation. In all panels, the results are an average of 9 independent replicates and error bars represent 95% confidence intervals for the sample mean.

We performed a DNase I susceptibility assay by incubating this enzyme for 24 h with R36A and R36AP in conditions allowing biofilm formation. A gradient of DNase I concentrations, ranging from 0.5 to 50 µg/ml was used. Biofilm biomass quantification indicates that DNase I reduces biofilm formation in a dose dependent way (Fig. 3A). As expected, the biomass reduction is directly related to a decrease of viable cells in the biofilm (data not shown). The effect of DNase I is similar in R36A and R36AP biofilms, however, R36AP biofilms always show a higher biomass than R36A biofilms at all tested DNase I concentrations, suggesting that R36AP biofilms are richer in eDNA. In fact, the biomass of the R36AP biofilm incubated with 0.5 µg/ml of DNase I, is similar to that of the R36A biofilm incubated in the absence of DNase I, indicating that the presence of this enzyme reduced the extra eDNA present in the R36AP matrix, resulting in a biofilm similar to that of R36A. Taken together, the data enable an argument to be made for the beneficial effect of lysogenic phages in biofilm development due to an increased presence of eDNA in the matrix.

To further explore this potential role of eDNA on biofilm development, we decided to determine the effect of the addition of external DNA to the medium since the time of seeding, on biofilm mass measured at 24 h of growth. DNA was extracted from the R36A strain (homologous DNA) and used at a final concentration of 10, 100 and 1000 ng/ml. To rule out any specific effect of pneumococcal DNA, the same experiments were repeated using DNA isolated from salmon sperm (heterologous DNA). As shown in Fig. 3B, incubation with DNA since biofilm seeding enhances biofilm development in a dose dependent manner, with a significant effect detected with as little as 10 ng/ml. This biomass increase parallels the number of viable cells in the biofilm (data not shown). Moreover, this effect is observed with both homologous and heterologous DNA, indicating that this was due to an intrinsic property of the DNA molecule and independent of the exact nucleotide sequence and donor organism.

Microscopy was used to explore the differences between untreated R36AP biofilms and those treated with 50 µg/ml of DNase I and 1000 ng/ml of DNA. In agreement with the results obtained by biomass quantification, treatment with DNase I resulted in sparser and thinner biofilms when compared to control (Fig. 2A and 2D). On the other hand, supplementation of the medium with DNA resulted in a more densely packed and thicker biofilm (Fig. 2A and 2C). These results further support that the limited lysis promoted by lysogenic phages during biofilm development leads to higher eDNA release resulting in stronger biofilm growth.

Due to the different kinetics of biofilm development of the lysogenic and non-lysogenic strains, we wanted to clarify if the role of DNA was only critical in the initial steps of biofilm establishment (initial cell attachment) or if its presence was necessary throughout the subsequent early phases of biofilm development. To this end, the wells where the biofilms were grown were pre-coated with DNA followed by incubation of the bacteria in DNA-free medium. After 24 h, biofilm mass was similar to the uncoated control (Fig. 3B), indicating that the observed DNA effect is not related with the initial adherence process.

Furthermore, we also examined whether DNA acts as a structural component of the biofilm or if the availability of extra nutrients, due to the presence of DNA in the medium, could explain the enhanced biofilm development. With that in mind, we grew biofilms in the presence of sonicated DNA and compared them with biofilms formed in the presence of intact DNA, using both homologous and heterologous DNA. In the presence of fragmented DNA, biofilm development assessed at 24 h was similar to that of biofilms grown in the absence of DNA and substantially less to that observed with intact DNA (Fig. 3B). This data revealed that large DNA fragments were essential for the enhancement of biofilm growth and suggested that DNA had an important structural role in biofilm architecture.

**Figure 4:**
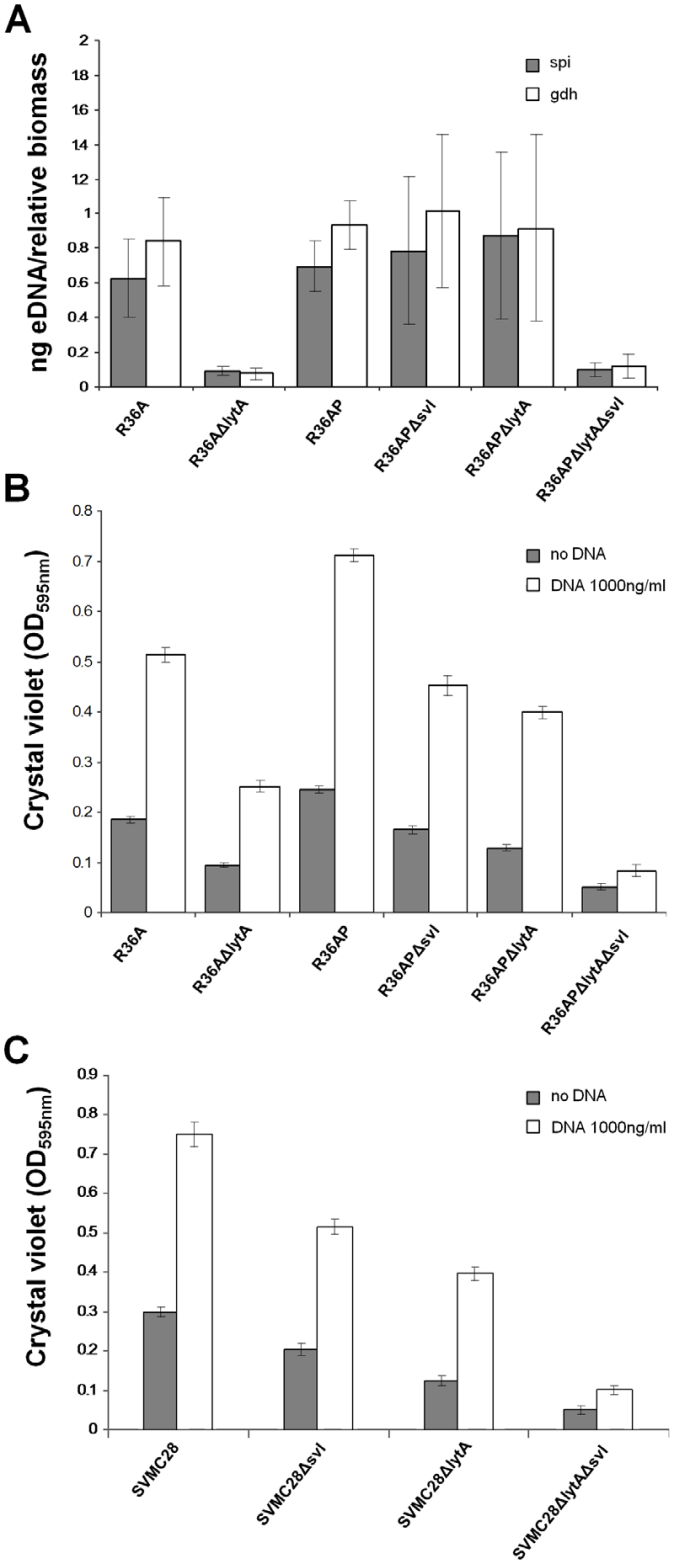
eDNA quantification and DNA impact on biofilm mass. **A**) Extracellular DNA was isolated from the biofilm matrices of R36A, R36AΔ*lytA*, R36AP, R36APΔ*svl*, R36APΔ*lytA* and R36APΔ*lytA*Δ*svl* and quantitative real-time PCR of two chromosomal genes, *spi* and *gdh*, was done. The relative biomass was quantified at OD_595 nm_ and the eDNA measurements were normalized to total biofilm mass. **B**) The effect of salmon sperm DNA (1000 ng/ml) added from seeding on biofilm biomass at 24 h was tested. R36APΔ*svl*, R36APΔ*lytA*, R36AΔ*lytA* and R36APΔ*lytA*Δ*svl* are mutants in which the phage lysin (Svl), the bacterial autolysin (LytA) or both were deleted. **C**) The same experiments described in panel B were done with the encapsulated wild type host of phage SV1, strain SVMC28, and its mutants. SVMC28Δ*svl*, SVMC28Δ*lytA* and SVMC28Δ*lytA*Δ*svl* are mutants in which the phage lysin (Svl), the bacterial autolysin (LytA) or both were deleted. In all panels, the results are an average of 9 independent replicates and error bars represent 95% confidence intervals for the sample mean.

To confirm if a higher eDNA release due to phage spontaneous induction is related to a strong biofilm development we determined the actual eDNA released into the biofilm of each strain, after 24 h of growth, by quantitative real-time PCR. Strains with a higher capacity to form biofilm (R36AP, R36A, R36APΔ*svl* and R36APΔ*lytA*) contain significantly more eDNA in comparison to the almost undetectable levels present in R36APΔ*lytA*Δ*svl* and R36AΔ*lytA*, two strains with poor biofilm forming capacity (Fig. 4A). The marked difference observed between lytic and non-lytic strains suggest that lytic events resulting in eDNA release have a strong positive impact in biofilm development.

DNA release upon phage induction is dependent on lysis. So, we reasoned that the addition of external DNA to biofilms of the mutant strains R36APΔ*svl*, R36APΔ*lytA* and R36APΔ*lytA*Δ*svl* would allow the development of more robust biofilms. Indeed, when the mutant strains were given exogenous DNA, biofilm development was strongly increased in R36APΔ*svl* and R36APΔ*lytA* (Fig. 4B). The addition of a large excess of DNA overcomes the impairments created by the ablation of either the phage or bacterial lysins, with the formation of more biofilm in the presence of DNA by these mutants than that observed when R36AP, where both lysins are functional, was incubated in the absence of exogenous DNA (Fig. 4B). As pointed out previously, the mutant lacking both lysin activities (R36APΔ*lytA*Δ*svl*) was incompetent to form stable biofilms and, even in the presence of excess DNA, failed to recover to the R36AP level. Thus the addition of DNA does not fully overcome the abolishment of the two major lysins present in R36AP. This is in contrast to R36AΔ*lytA* that responds well to the addition of external DNA. Although both mutants present similar amounts of eDNA (Fig. 4A), the R36AΔ*lytA* strain forms more biofilm biomass than strain R36APΔ*lytA*Δ*svl* (Fig. 1A) and this effect is even more pronounced in the number of viable bacteria in the biofilm (Fig. 1B). It has been previously shown that even when phage and bacterial lysins are deleted, phage induction decreases cell viability as phages express holins that collapse the cell membrane potential resulting in host cell death [32]. Thus, this difference in cell viability between R36AΔ*lytA* and R36APΔ*lytA*Δ*svl* may be sufficient to compromise the enhancement of biofilm development in the presence of added DNA observed in the later strain.

To test if the presence of a capsular polysaccharide could influence our results, we characterized the behavior of strain SVMC28 and its mutants in both phage and bacterial lysins. SVMC28 is an encapsulated strain and the natural host of the SV1 phage. The results obtained were superimposable to those of strain R36AP and its mutants, with the same relative biomass produced by the parental strain and its mutants in the absence of DNA and the same effect seen upon DNA addition (Fig. 4C). This indicates that our observations were reproducible in different genetic backgrounds and, more importantly, that the capsule did not qualitatively alter our conclusions Overall, our results indicate that the release of eDNA through controlled lytic events is a key factor for biofilm formation in *S. pneumoniae* and that lysogenic phages are important adjuvants for its incorporation in the biofilm matrix independently of the presence of a capsular polysaccharide.

## Discussion

Prophages are extremely common among *S. pneumoniae* isolates causing infections in humans [24]. The lysogenic lifestyle results in the establishment of the phage genome inside the bacterial host where it can remain in a dormant state replicating together with the bacterial chromosome. An important feature is the possible transition from the repressed lysogenic state to lytic development, that ultimately leads to host cell death and release of the newly produced phage particles. Prophage induction can occur spontaneously in a fraction of the lysogenic bacterial population or massively upon external stimuli [26], [27].

Here we investigated the impact of lysogeny in *S. pneumoniae* biofilm formation exploring its role in the early development of these structures. Our data provided evidence that prophage carriage had a positive impact on pneumococcal biofilm formation through spontaneous induction of the lytic cycle. Phage induction results in the death of their bacterial hosts, however we showed that this phage-mediated lysis enhances biofilm formation, suggesting that in this context the bacterial population as a whole could benefit from limited prophage induction. Studies on gene expression in biofilms of various species have identified phage genes as overexpressed relative to planktonic growth while other studies showed the existence of lysis inside biofilms and proposed that it could increase biofilm fitness [35]–[40]. Our results corroborate this previous proposal in the context of *S. pneumoniae* biofilms, clearly identifying the phage activated lytic machinery as a key player in this effect. Interestingly, phage mediated bacterial lysis within the biofilm has also been described in other bacterial species. However, in contrast to our study, in those cases, phage induction results in the death of a large fraction of the bacterial population and occurs in the later stages of biofilm development [36], [38]–[40].

The impact of lysogenic phages on pneumococcal populations is still an open question since comparative genomic analysis did not reveal any phage-encoded virulence factors, contrary to other related streptococcal pathogens such as *Streptococcus pyogenes* [41], [42]. The observed biofilm potentiating role of lysogenic phages and the proposed importance of these structures in colonization [11] could explain in part the high incidence of lysogeny in *S. pneumoniae* natural populations [24], [25]. Furthermore, a high frequency of lysogeny is characteristic of many bacterial pathogens [43] as well as of bacterial populations in the environment [44], raising the possibility that the influence of lysogeny on the ability of pneumococci to form biofilms could be paralleled in other bacterial species.

The mechanism by which spontaneous prophage-mediated cell lysis leads to increased biofilm development was also addressed in this study. We gathered evidence that DNA released through this process to the extracellular environment contributes to biofilm formation in *S. pneumoniae*. An approximately six-fold increase in eDNA was detected in strains carrying prophages and functional bacterial or phage lysins. These lysogenic strains were also characterized by forming biofilms with a higher biomass and cell viability. This role of eDNA is consistent with previous findings in this species, although in those studies the source of eDNA was not identified [6], [23]. We observed that eDNA is not involved in the initial attachment stage, since pre-treatment of the plastic substrate with DNA did not increase biofilm formation. In agreement, a high concentration of DNAse I added from the onset of biofilm incubation still allowed bacterial surface attachment and biofilm formation, although in these conditions bacteria failed to form the thick and dense structures observed in the absence of DNase I. To our knowledge, this is the first study of the role of eDNA in initial adhesion of pneumococcal cells to a surface. Although in some bacterial species eDNA plays an important role in this initial step [16], [45], similar results to ours were already observed with another Gram-positive bacterium [15]. Being such a complex lifestyle, it is plausible that in different microorganisms the importance of the various mechanisms for biofilm establishment is also different. The factors or substances that promote initial attachment remain to be identified in *S. pneumoniae*. However, eDNA played an important role already in the early stages of biofilm development, since spontaneous phage induced lysis is detected in the early hours of biofilm establishment and the R36AP lysogen showed a more robust biofilm development at all time points. Accordingly, a mutant lacking the phage lysin produced less biofilm and in a delayed fashion, a behaviour that was similar to the mutants lacking the major bacterial autolysin LytA. Both observations are consistent with a possibly slower accumulation of eDNA in the matrix and with an important role of eDNA at various stages of biofilm formation.

Our data indicates that eDNA is an important structural component of *S. pneumoniae* biofilms, ensuring stability of the overall architecture of these structures. Although DNAse I treatment resulted in eDNA degradation with the consequent reduction in biofilm formation, the critical result that definitely establishes this structural role of DNA was the observation that addition of fragmented DNA did not affect biofilm development, whereas intact DNA led to increases in both mass and bacterial viability in biofilms, indicating that the long strands of DNA may allow more intercellular cohesion thereby increasing biofilm stability. These results are supported by studies in other species that have proposed DNA as an essential component of the extracellular polymeric substance that constitutes the biofilm matrix [13], [15], [16], [23], [46]. Thus, cell lysis mediated by lysogenic phages influences the matrix composition, thereby contributing to the pneumococcal biofilm structural stability. Since spontaneous phage induction occurs in different areas of the biofilm, it is expected to contribute significantly to the abundance and widespread localization of eDNA.

In contrast to limited cell lysis due to spontaneous phage induction, massive phage induction in the presence of an external inducing agent could disrupt biofilms drastically, an hypothesis supported by the use of lytic phages as powerful anti-biofilm agents active against different microorganisms [47], [48]. In fact, preliminary results from our group indicate that Mitomycin C phage induction is able to disrupt to a large extent biofilms of lysogenic strains. If the proportion of induced cells is large, more cells lyse than are contributing to the biofilm resulting in an overall loss of biofilm mass. This is in agreement with the natural biofilm demise mediated by substantial phage induction proposed for some *Pseudomonas aeruginosa* strains that facilitate differentiation and dispersal of biofilm associated bacteria [36], [39], [40]. The beneficial or detrimental effect of prophage induction on biofilm formation seems to be quantitatively regulated by the proportion of lysogenic bacteria undergoing lytic induction.

In conclusion, we showed that limited activation of prophages into the lytic cycle, thereby promoting host lysis and eDNA release, contributes to enhanced pneumococcal biofilm production. This more efficient biofilm development afforded by lysogenic phages may be an important aspect in the biology of the bacteria since lysogeny is highly prevalent in pneumococci. Our data provided new insights into the factors that influence the formation and maintenance of biofilms whose occurrence and importance *in vivo* is increasingly recognized.

## Materials and Methods

### Bacterial strains, culture conditions and DNA manipulations

Bacterial strains SVMC28 and R36A were obtained from the Rockefeller University collection (A. Tomasz). R36A is a non-lysogenic, non-encapsulated strain [24]. SVMC28 is an encapsulated (serotype 23F) clinical isolate, lysogenic for phage SV1 encoding the Svl phage lysin [32]. R36AΔ*lytA* was kindly provided by S. Filipe. SVMC28 derived mutants SVMC28Δ*svl*, SVMC28Δ*lytA* and SVMC28Δ*svl*Δ*lytA* belong to the Faculdade de Medicina de Lisboa collection. The SV1-lysogenized strains R36AP, R36APΔ*lytA*, R36APΔ*svl* and R36APΔ*lytA*Δ*svl* are also from the Faculdade de Medicina de Lisboa collection. All strains were described previously [32]. All *S. pneumoniae* strains were grown in a casein-based semi-synthetic medium (C+Y) at 37°C without aeration or in tryptic soy agar (TSA) (Oxoid, Hampshire, England) supplemented with 5% (v/v) sterile sheep blood incubated at 37°C in 5% CO_2_. For overnight cultures, pneumococcal mutant strains were grown in the presence of 2 µg/ml erythromycin or 4 µg/ml chloramphenicol (Sigma, Steinheim, Germany) or both, as appropriate. After selective growth, the culture was diluted 1∶100 in fresh medium and grown until the appropriate optical density. Chromosomal DNA from *S. pneumoniae* strain R36A was isolated following previously described procedures [49]. Sperm salmon DNA was purchased from Invitrogen Co. (Carlsbad, California, USA).

### Biofilm biomass quantification

Biofilm formation was determined by the ability of cells to grow adherent to 96-well flat-bottom polystyrene microtiter plates (Nunc^TM^, Roskilde, Denmark) in static conditions. Cells were grown in C+Y medium, with selective antibiotic when necessary, to an optical density measured at 600 nm (OD_600nm_) between 0.5 and 0.6 and then diluted 1∶4 in fresh medium to a final volume of 200 µl per well. Microtiter plates were incubated at 37°C and biofilm mass was determined by staining with crystal violet [23] and measuring the OD_595nm_ using a plate reader (Tecan Infinite M200 with i-control^TM^ software V1.40). The incubation times at which the biomass was quantified were selected based on preliminary experiments in order to monitor the dynamics of biofilm growth and dispersal. Shorter time intervals were selected when biofilm mass showed steeper variations. The incubation times at which biomass was quantified were 6 h, 12 h, 18 h, 20 h, 24 h, 26 h, 28 h and 30 h of incubation. A control with only C+Y medium was also done for all time points and the values were subtracted to those measured for all strains.

Quantitative determination of biofilm formation was also evaluated in the presence of desoxiribonuclease I (DNase I) and DNA, incorporated in the medium. DNase I (Sigma, Steinheim, Germany) was used at a final concentration of 0.5, 5 or 50 µg/ml and biofilm mass was measure after 24 h of incubation. DNA from R36A strain or salmon sperm was added at 10, 100 or 1000 ng/ml to the medium and determination of biofilm formation was carried out 24 h post incubation. Values obtained from medium supplemented with DNAse I and DNA were subtracted in all strains. To test if DNA was important in biofilm adherence, the plate wells were incubated with 1000 ng/ml of R36A DNA overnight at 4°C to condition the plastic surface. The solution was then discarded and the biofilm was seeded as described before. Biofilm mass was determined at 24 h post incubation. To determine if the impact of DNA on biofilm formation was due to a structural role, DNA from R36A was broken by sonication for 5 min at 0.63 A and 50–60 Hz in a Transsonic T570 (Elma, Germany), and added to the medium at 1000 ng/ml. DNA fragmentation was confirmed by agarose gel electrophoresis. Biofilm formation was compared to biofilms grown in the presence of 1000 ng/ml of intact DNA.

### Biofilm colony forming units (CFU) assays

Biofilms were grown in 96-well plates at 37°C as described for the biofilm biomass quantification assay. CFUs were determined at the selected time points between 6 h and 30 h of incubation. Liquid medium with bacteria was gently removed from the wells, which were washed twice with phosphate buffered saline (PBS) 1×, pH 7.2 (Invitrogen, Grand Island, New York) to eliminate unbound bacteria without disturbing the adherent biofilm. 200 µl of PBS were then added to each well and biofilms were scraped thoroughly, including well edges. The well contents were recovered and the total CFU number was determined by serial dilution and plating on appropriate media.

To test the effect of DNase I and DNA on biofilm development, DNase I was added to the growth medium to a final concentration of 0.5, 5 or 50 µg/ml. After 24 h of incubation at 37°C, CFUs were determined as described above. When using DNA to evaluate its effect on biofilm formation, DNA from salmon sperm was added to the growth medium at a final concentration of 1000 ng/ml and CFUs were determined as described above.

### Phage plaque assays

Plaque assays were performed as described elsewhere [32].In detail, basal plates were made by pouring C+Y medium with 170 U catalase per ml and 1% agar into Petri dishes. A lawn culture of R36A strain grown to an OD_600nm_ of 0.2 was mixed with soft agar containing C+Y supplemented with 170 U catalase per ml and 0.35% agar. The entire mixture was spread onto basal plates. After hardening, phage preparations were applied in 10 µL aliquots directly on the soft agar with the R36A indicator strain. Incubation was performed at 30°C for 18 h. To obtain the phage preparation, at the chosen time points after biofilm seeding each well was scraped thoroughly including well edges. The harvested biofilms were filtered through a 0.45 µm-pore-size membrane followed by filtering with a 100 000 MWCO polyethersulfone membrane (Vivaspin concentrator, Sartorius Stedim biotech, Goettingen, Germany), that retains and concentrates the SV1 phage [32]. The phage concentrate was stored at 4°C for a maximum of 24 h until usage. The filtrate containing proteins <100 KDa, that could cause bacterial lysis such as LytA and bacteriocins, was also used to eliminate the possibility that lysis of the indicator strain was caused by bacterial products and not caused by phage infection. Images of the plates were acquired with the high-performance stereo-microscope Leica MZ7.5 (Leica Microsystems, Germany) and the number of plaque forming units (PFUs) was counted manually by visual inspection of the image.

### Confocal laser scanning microscopy (CLSM)

Biofilms were stained by using a Live/Dead BacLight bacterial viability kit (Invitrogen, Carlbad, USA) and examined by CLSM. Syto9/PI labeled biofilms allowed for monitoring the viability of bacterial populations as a function of the membrane integrity of the cell. Cells with a compromised membrane (dead cells) will stain red whereas cells with an intact membrane (live cells) will stain green. Whenever DNA and DNase I effects were tested, the medium was supplemented before biofilm seeding (t = 0). In all experiments, biofilms were analyzed after 24 h of incubation. Images were acquired on a Zeiss LSM510 META confocal microscope (Carl Zeiss, Jena, Germany) using a PlanApochromat 63×/1.4 objective for cell viability assays and a C-AproChromat 40×/1.2. Syto 9 fluorescence was detected using the 488 nm laser line of an Ar laser (45 mW nominal output) and a BP 505–550 filter. PI fluorescence was detected using a DPSS 561 nm laser (15 mW nominal output) and a LP 575 filter. For imaging, the laser power was attenuated to 1–2% of its maximum value. The pinhole aperture was set to 1 Airy unit.

#### Purification and quantification of eDNA

Biofilms were grown in 96-well plates at 37°C as reported above. eDNA was purified from 24 h biofilms exactly as previously described [50]. eDNA was quantified by real-time PCR using the primes gdh-up (5′-ATGGACAAACCAGCNAGYTT) and gdh-dn (5′-GCTTGAGGTCCCATRCTNCC) and spi-up (5′-TTATTCCTCCTGATTCTGTC) and spi-dn (GTGATTGGCCAGAAGCGGAA), amplifying the *gdh* and *spi* genes used for multilocus sequence typing (MLST), respectively. These are housekeeping genes located far apart in the R36A chromosome. PCRs were performed on non-diluted samples with the SYBR Green Jump Start Taq Ready Mix (Sigma, Steinheim, Germany), according to the manufactureŕs recommendations. Purified R36A genomic DNA at known concentrations was also subjected to quantitative real-time PCR with each primer pair to generate a standard curve used to calculate the concentration of eDNA in the unknown samples. PCR was performed in a 7500 Fast Real-Time PCR System (Applied Biosystems, Life Technologies, Carlsbad, California, USA). To account for potential differences in biomass, the average OD_595nm_ of each biofilm was determined and used to calculate the relative OD_595nm_ of each biofilm with respect to the OD_595nm_ of the wild type R36A biofilm. The nanogram of eDNA per relative biomass of each biofilm was then calculated by dividing its total eDNA (ng) by its relative OD_595nm_.
